# Reduced Plasma Ascorbate and Increased Proportion of Dehydroascorbic Acid Levels in Patients Undergoing Hemodialysis

**DOI:** 10.3390/life11101023

**Published:** 2021-09-28

**Authors:** Yuta Doshida, Mitsuyo Itabashi, Takashi Takei, Yuka Takino, Ayami Sato, Wako Yumura, Naoki Maruyama, Akihito Ishigami

**Affiliations:** 1 Molecular Regulation of Aging, Tokyo Metropolitan Institute of Gerontology, Tokyo 173-0015, Japan; doshida-yuta@ed.tmu.ac.jp (Y.D.); mitsuyo_itabashi@tmghig.jp (M.I.); takashi_takei@tmghig.jp (T.T.); takino_y@tmig.or.jp (Y.T.); ayamis@tmig.or.jp (A.S.); wyumura@iuhw.ac.jp (W.Y.); mars610jp@aol.com (N.M.); 2 Department of Biological Sciences, Tokyo Metropolitan University, Tokyo 192-0397, Japan; 3 Department of Nephrology, Tokyo Metropolitan Geriatric Hospital, Tokyo 173-0015, Japan; 4 Department of Nephrology and Endocrinology, Tohoku Medical and Pharmaceutical University Hospital, Miyagi 983-8512, Japan; 5 Saitama Central Hospital, Saitama 354-0045, Japan

**Keywords:** chronic kidney disease, hemodialysis, hyperkalemia, ascorbate, dehydroascorbic acid, vitamin C, potassium

## Abstract

Ascorbate functions as an electron donor and scavenges free radicals. Dehydroascorbic acid (DHA), the oxidized form of ascorbate, is generated as a result of these reactions. While low plasma ascorbate levels have been reported in hemodialysis patients worldwide, no studies have measured DHA because it is not generalized. In this study, we aimed to clarify whether plasma ascorbate levels are low in dialysis patients and whether plasma ascorbate levels fluctuate before and after dialysis. Moreover, we applied our previously established method to measure the plasma ascorbate and DHA levels in chronic kidney disease (CKD) stage G3–G5 non-hemodialysis-dependent patients, and pre- and post-dialysis plasma ascorbate and DHA levels in CKD stage G5D hemodialysis patients. The sample size was calculated using G-power software. The pre-dialysis plasma total ascorbate levels, including DHA, were significantly (56%) lower in hemodialysis patients than in non-hemodialysis-dependent CKD patients. After dialysis, there was a 40% reduction in the plasma total ascorbate levels. Hemodialysis increased the post-dialysis plasma proportions of DHA from 37% to 55%. The study results demonstrated lower plasma total ascorbate levels in hemodialysis patients compared with in non-hemodialysis-dependent CKD patients; these low levels in hemodialysis patients were further reduced by hemodialysis and increased DHA proportion.

## 1. Introduction

Vitamin C (L-ascorbic acid) is a water-soluble micronutrient and antioxidant that scavenges reactive oxygen species, such as superoxide and hydroxyl radicals [1,2,3]. Under physiological pH conditions, ascorbic acid most commonly exists in its mono-anion form, ascorbate [4]. In addition to its antioxidant property, ascorbate also contributes to numerous well-defined enzymatic reactions, involving collagen hydroxylation, norepinephrine biosynthesis, tyrosine metabolism, and peptide hormone amidation [5,6,7]. Dehydroascorbic acid (DHA), an oxidized form of ascorbic acid, is generated as a result of these reactions, and an increase in the DHA proportion suggests that the oxidative stress levels in vivo may be increasing [8].

Many vertebrates can synthesize ascorbate from glucose de novo in the liver [9]. However, primates, including humans, cannot synthesize ascorbate because they carry multiple mutations in the *Gulo* gene encoding L-gulono-γ-lactone oxidase, the last enzyme in the ascorbate biosynthesis pathway [10]. Therefore, humans must consume ascorbate from dietary sources, such as fresh fruits and vegetables, to prevent scurvy. Scurvy is a condition resulting from insufficient ascorbate in the body. Most scurvy symptoms, including anemia, weakness, and gingival bleeding, also often occur in hemodialysis patients [11].

In recent years, the increasing number of patients undergoing dialysis has become a social problem worldwide. The mean age of incident dialysis has increased among patients aged ≥45 years, especially among those aged ≥65 years [12]. In Japan, 65.5% of patients undergoing dialysis were aged ≥65 years at the end of 2012, indicating an increased incidence of dialysis in the aging population [12].

Low plasma ascorbate levels have long been reported globally in some hemodialysis patients [13,14,15,16,17,18,19]. However, the reasons remain unclear, although hemodialysis patients have dietary restrictions such as limited protein, salt, and potassium intake. The consumption of fruits and vegetables containing high amounts of ascorbate is also restricted due to their high potassium content.

This prospective study aimed to confirm whether patients on hemodialysis had low plasma ascorbate and DHA levels compared with those in non-hemodialysis-dependent patients with chronic kidney disease (CKD). We also examined whether dialysis reduced plasma ascorbate and DHA levels and the percentage of DHA per total ascorbic acid, including DHA. Finally, we evaluated whether any clinical test items showed a causal relationship with plasma ascorbate and DHA levels in patients on hemodialysis.

## 2. Materials and Methods

### 2.1. Study Patients

The sample size was calculated using G-power software [20]. The recruit of patients for analysis was executed without bias. We aimed for a total sample size of *n* = 44 as this would yield sufficient power (80%) to detect large effects size (d = 0.9) in a two-sided Wilcoxon signed-rank test at an alpha-rate of 5%. This study recruited a total of 53 CKD patients, consisting of CKD stage G3–G5 (mean estimated glomerular filtration rate (eGFR) 23 mL/min/1.73 m^2^; *n* = 34) and CKD stage G5D hemodialysis (*n* = 19) who had been regularly visiting an outpatient clinic of the Tokyo Metropolitan Geriatric Medical Center between October 2018 and December 2019. The grade of renal dysfunction was determined based on the guidelines of the National Kidney Foundation Kidney Disease Outcomes Quality Initiative [21]. In addition, of the 19 CKD stage G5D hemodialysis patients, 10 underwent maintenance hemodialysis and 9 underwent hemodiafiltration. The procedures were performed three times weekly for 9–12 h per week, at a blood flow rate of 150–200 mL/min and dialysis flow rate of 500 mL/min using dialyzers with a surface area of 1.1–2.1 m^2^. The dialysate sodium concentration was 140 mEq/L and the potassium concentration was 2.0 mEq/L. The mean duration of dialysis therapy was 3.6 years. The mean Kt/V was 1.3.

### 2.2. Collection of Blood and Urine Samples

Blood samples for the measurement of clinical test items and total ascorbate, including DHA, were obtained at the same time. For the determination of ascorbate and DHA levels, blood samples were drawn into VENOJECT^®^ collection tubes (Terumo Corporation, Tokyo, Japan) containing ethylenediaminetetraacetic acid (EDTA)-2Na as an anticoagulant. Furthermore, blood samples from hemodialysis patients were drawn from the arteriovenous fistula or catheter before the start of dialysis (pre-dialysis sample) and immediately after ending the dialysis period (post-dialysis sample). All the following procedures were performed within 2 h after sampling, as we had previously confirmed that the ascorbate values were unstable beyond 2 h [22]. The plasma was obtained by centrifugation at 1700× *g* for 10 min. After the plasma was collected, 0.5 mL of supernatant was immediately mixed with 0.5 mL of cold 10% metaphosphoric acid (FUJIFILM Wako Pure Chemical Corporation, Osaka, Japan) containing 1 mmol/L EDTA (Dojindo Laboratories, Kumamoto, Japan), and was centrifuged at 21,000× *g* for 15 min at 4 °C to measure the ascorbate levels. After collecting the urine samples, 0.5 mL of urine was immediately mixed with 0.5 mL of cold 10% metaphosphoric acid containing 1 mmol/L EDTA and centrifuged at 21,000× *g* for 15 min at 4 °C to measure the ascorbate levels. All of the samples were stored at −80 °C until use.

### 2.3. Determination of Ascorbate and DHA Levels

Ascorbate and DHA levels were measured using high-performance liquid chromatography and electrochemical detection as previously described [8]. After thawing, the plasma and urine samples were centrifuged at 21,000× *g* for 10 min at 4 °C. To determine the total ascorbate, including DHA, the centrifugal supernatants were reduced with tris(2-carboxyethyl)phosphine hydrochloride for 2 h on ice. After reduction, the reaction mixture was diluted with 5% metaphosphoric acid containing 0.5 mmol/L EDTA and was analyzed for the total ascorbate using high-performance liquid chromatography coupled with electrochemical detection. Separation was performed using an Atlantis dC18 5-μm column (4.6 × 150 mm) combined with an Atlantis dC18 5-μm guard column (4.6 × 20 mm) from Nihon Waters (Tokyo, Japan). The mobile phase comprised 50 mM phosphate buffer (pH 2.8), 540 μM EDTA, and 2% methanol at a flow rate of 1.3 mL/min. The electrical signals were recorded using an electrochemical detector with a glassy carbon electrode at +0.6 V. Electrical signal data from the electrochemical detector were collected using Waters Empower 3 software (Nihon Waters). The DHA value was determined by subtracting the ascorbate value from the total ascorbate value. The level of ascorbate in the urine was immediately affected after a meal. Therefore, we evaluated the plasma ascorbate levels in the samples with urinary ascorbate <0.5 mM.

### 2.4. Study Items

Data on patient age, sex, and results of clinical investigations, that is, white blood cell count; hematocrit, platelet count; hemoglobin concentration total protein concentration; total iron-binding capacity (TIBC); and levels of albumin, C-reactive protein, aspartate aminotransferase (AST), blood urea nitrogen, creatinine, uric acid, sodium, potassium, calcium, phosphorus, triglyceride, total cholesterol, low-density lipoprotein (LDL) cholesterol, iron, ferritin, β2-microglobulin, prealbumin, hemoglobin A1c (HbA1c), and parathyroid hormone were collected from the medical records at the time of blood sampling.

### 2.5. Statistical Analysis

The results and clinical characteristic data are expressed as means ± standard deviation (SD). The probability of statistical differences between experimental groups was determined using Wilcoxon rank-sum and Wilcoxon signed-rank tests. We verified that the Kendall’s rank correlation coefficients between the ascorbate and DHA concentration and clinical characteristics were not zero. Statistical differences were considered significant at *p* < 0.05. Calculation of *q*-values on a multiple test were made using the Benjamini−Hochberg method.

## 3. Results

### 3.1. Clinical Characteristics of Patients with CKD Stage G3–G5 and Those Undergoing Hemodialysis

A total of 53 CKD patients, consisting of 34 CKD stage G3–G5 and 19 CKD stage G5D hemodialysis patients, were enrolled. Seven CKD stage G3–G5 patients with urine ascorbate levels > 0.5 mM just before collecting the blood and urine, suggesting a supplemental intake of ascorbate, were excluded from the analysis. Therefore, the results of 27 CKD stage G3–G5 patients were used for the analysis. The clinical characteristics of all 46 CKD patients are shown in Table 1. The blood urea nitrogen, creatinine, and β2-microglobulin levels were outside of the normal ranges and significantly differed between patients with CKD stage G3–G5 and those undergoing hemodialysis (bold in Table 1).

### 3.2. Plasma Ascorbate and DHA Levels in CKD Stage G3–G5 and Hemodialysis Patients

**Figure 1:**
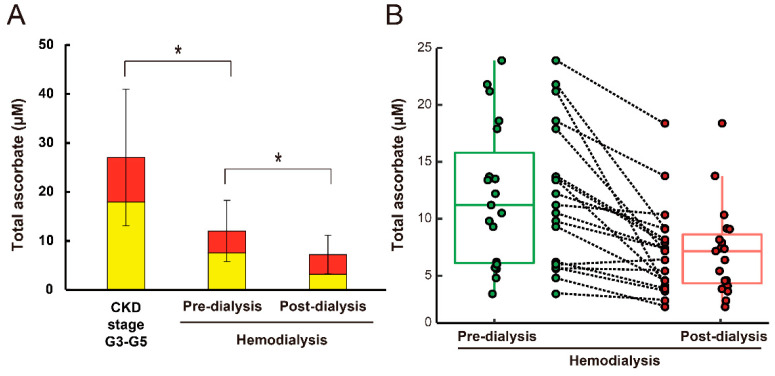
(**A**) Plasma total ascorbate, ascorbate, and dehydroascorbic acid (DHA) levels of patients. Ascorbate (yellow column) and DHA (red column) levels were determined as described in the Materials and Methods. Chronic kidney disease (CKD) stage G3–G5 patients, *n* = 27. Hemodialysis patients, *n* = 19. Values are expressed as mean ± SD of the total ascorbate. * *p* < 0.05. (**B**) Dots in boxplots express the total ascorbate levels. Center lines are the median values of each group. Individual pre- and post-dialysis plasma total ascorbate levels are shown in the middle.

The plasma total ascorbate, including DHA, ascorbate, and DHA levels, were measured (Figure 1 and Table A1). The pre-dialysis plasma total ascorbate and ascorbate levels in the hemodialysis patients were significantly lower by 56% and 58%, respectively, than those in patients with CKD stage G3–G5. Similarly, pre-dialysis plasma DHA levels were significantly (by 50%) lower in hemodialysis patients than in CKD stage G3–G5 patients. The percentages of DHA per total ascorbic acid were 34% and 37%, respectively, in CKD stage G3–G5 and hemodialysis patients. In addition, the individual pre- and post-dialysis plasma total ascorbate levels in hemodialysis patients are shown in Figure 1B.

### 3.3. Pre- and Post-Dialysis Plasma Ascorbate and DHA Levels in Hemodialysis Patients

After dialysis, the plasma total ascorbate, ascorbate, and DHA levels were reduced by 40%, 57%, and 11%, respectively (Figure 1A and Table A1). Moreover, the percentage of DHA per total ascorbic acid increased to 55% in post-dialysis plasma.

### 3.4. Relationships between Clinical Characteristics and Plasma Ascorbate and DHA Levels

**Figure 2:**
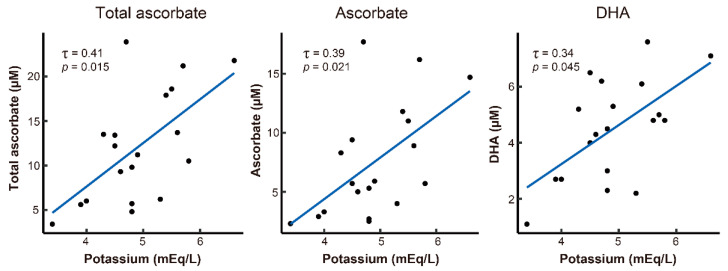
Scatterplots of the plasma potassium levels and total ascorbate, ascorbate, and dehydroascorbic acid (DHA) levels. The dots indicate the expressed data of individual patients on hemodialysis (*n* = 19). The blue lines are regression lines. The Kendall’s rank correlation coefficients are shown in Table 2.

We then analyzed the relationships between the clinical characteristics and the pre-dialysis plasma total ascorbate, ascorbate, and DHA levels in the hemodialysis patients. An analysis of the Kendall’s rank correlation coefficient showed a trend of correlation between pre-dialysis total ascorbate, ascorbate, and DHA levels and plasma potassium levels (Figure 2 and Table 2). In addition, no patients were administered medications such as sodium or calcium polystyrene sulfonate for hyperkalemia. Moreover, pre-dialysis DHA levels also tended to correlate with plasma albumin and iron levels (bold in Table 2). However, no association was observed between the plasma total ascorbate, ascorbate, and DHA levels and the other clinical characteristics.

## 4. Discussion

The results of this study demonstrated low plasma total ascorbate, including DHA, ascorbate, and DHA levels, in hemodialysis patients compared with those in non-hemodialysis-dependent patients with CKD. Moreover, the low plasma total ascorbate, ascorbate, and DHA levels in hemodialysis patients were further reduced by a single hemodialysis treatment session. In addition, hemodialysis increased the DHA proportion, suggesting the oxidative stress levels in vivo may be increasing. We also found that plasma total ascorbate, ascorbate, and DHA levels in hemodialysis patients tended to correlate with plasma potassium levels. In general, hemodialysis patients are forced to restrict their diets, especially potassium-rich fruits and vegetables, so as to prevent hyperkalemia, which is a risk factor for dialysis morbidity and mortality [23]. Most of these fruits and vegetables also contain high amounts of ascorbate. Therefore, plasma ascorbate and DHA levels in hemodialysis patients might be correlated with plasma potassium levels.

**Figure A1:**
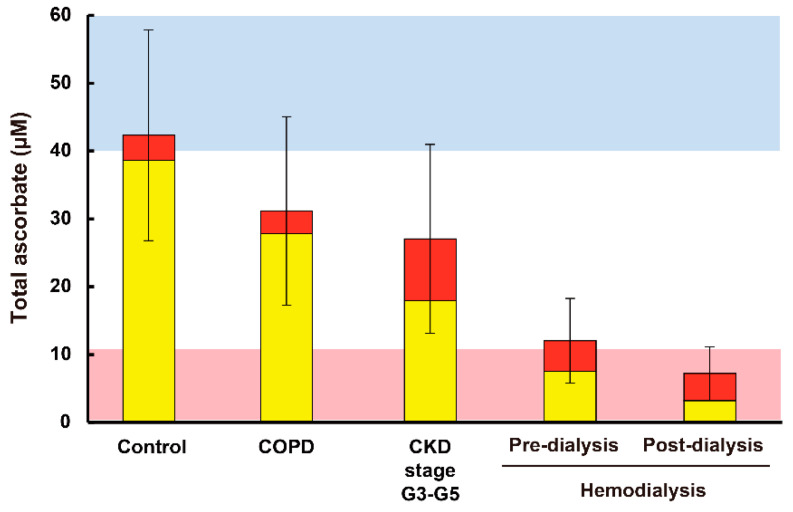
Plasma total ascorbate, ascorbate, and dehydroascorbic acid (DHA) levels. Healthy controls, *n* = 28 [22]. Chronic obstructive pulmonary disease (COPD) patients, *n* = 39 [22]. Chronic kidney disease (CKD) stage G3–G5 patients, *n* = 27. Hemodialysis patients, *n* = 19. Ascorbate (yellow column) and DHA (red column) levels are determined. The average concentration of ascorbate in the plasma of healthy humans is 40–60 μM (blue zone) [25,26]. There is a risk of developing scurvy when the plasma ascorbate concentration drops to below 11 μM (red zone) [25,26]. Values are expressed as mean ± SD.

The recommended dietary allowance (RDA) of vitamin C to prevent scurvy in healthy adults is 100 mg per day in Japan, and 90 mg and 75 mg per day for men and women, respectively, in the United States [24]. The average concentration of ascorbate in the plasma of healthy humans is 40–60 μM [25,26]. When the plasma ascorbate concentration drops to below 11 μM, there is a risk of developing scurvy, a threshold below which the levels are conventionally considered deficient [25,26]. Our previous study of chronic obstructive pulmonary disease (COPD) and plasma total ascorbate levels measured using the same procedure and method reported significantly lower plasma total ascorbate levels in patients with COPD than those in healthy elderly people (Figure A1) [22]. The plasma total ascorbate levels in patients with COPD and healthy elderly people were 31.2 ± 13.9 and 42.3 ± 15.5 µM, respectively. Furthermore, the observed plasma levels in patients with non-hemodialysis-dependent CKD and those undergoing hemodialysis in the present study were lower than those in patients with COPD (Figure A1). The plasma total ascorbate levels in patients with non-hemodialysis-dependent CKD and those undergoing hemodialysis were 27.1 ± 13.9 and 12.0 ± 6.2 µM, respectively (Figure A1). As there is a risk of developing scurvy at plasma ascorbate concentrations below 11 μM [25,26], many hemodialysis patients are likely to develop scurvy. Globally, many hemodialysis patients have developed scurvy [11,27,28].

Our comparisons of the percentages of DHA per total ascorbate in plasma among healthy elderly people, COPD patients, non-hemodialysis-dependent CKD patients, and hemodialysis patients showed notably higher percentages in patients with non-hemodialysis-dependent CKD (34%) and those undergoing hemodialysis (37%) compared with those in patients with COPD (11%) and healthy elderly people (9%) (Figure A1) [22]. The percentage of DHA per total ascorbate in the post-dialysis plasma of hemodialysis patients was high (55%), which may reflect increased oxidative stress levels in vivo after dialysis in hemodialysis patients, and may also accelerate aging, as oxidative stress may be a cause of aging. Wang et al. [17] reported that plasma ascorbate concentrations were reduced by a median of 33% following dialysis. Deicher et al. [29] also reported that hemodialysis caused a 50–75% decrease in plasma ascorbate levels. In the present study, the plasma total ascorbate levels were reduced to 40% by hemodialysis. Thus, hemodialysis reduces the plasma total ascorbate concentration in hemodialysis patients. However, there have been no reports concerning plasma DHA levels and DHA proportions in hemodialysis patients. The present study is the first of such a report in hemodialysis patients.

There has long been concern regarding the accumulation and deposition of oxalate with an increased intake of vitamin C, as oxalate is a breakdown product of vitamin C and is heavily excreted by the kidneys [30]. Oxalate crystallization occurs at >30 mM [31] and high plasma oxalate levels occur in hemodialysis patients [32,33,34]. A recent prospective case series exploring high-dose intravenous vitamin C (15–100 g) administration reported that increased vitamin C intake was not associated with any cases of symptomatic renal stones and kidney injury [35]. Moreover, no controlled trials, including the most recent VITAMIN randomized trial, have reported significant side effects of vitamin C [36].

CKD patients with higher levels of plasma calcium, phosphate, and parathyroid hormone have a high risk of death because CKD often causes abnormal calcium and phosphate metabolism and hyperparathyroidism [37,38,39,40,41]. Therefore, it is important to control the plasma calcium, phosphate, and parathyroid hormone levels in non-hemodialysis CKD and hemodialysis patients [42]. Through a systematic review and meta-analysis, Ke et al. [43] reported that vitamin C supplementation in CKD patients had no positive effect that influenced the plasma phosphate or parathyroid hormone levels, but it increased plasma calcium levels in the short term. In the present study, we did not detect any correlation between plasma ascorbate and plasma calcium, phosphate, and parathyroid hormone levels in hemodialysis patients. We observed a positive correlation tendency between the plasma total ascorbate, ascorbate, and DHA and plasma potassium levels in hemodialysis patients. Hemodialysis patients with dietary potassium restrictions to prevent hyperkalemia may limit their consumption of fresh vegetables and fruits rich in ascorbate. Therefore, hemodialysis patients may have low plasma total ascorbate, ascorbate, and DHA levels.

Recently, the increase in frailty in the elderly has become a global social problem. The Dialysis Morbidity and Mortality Wave 2 cohort study reported that >60% of patients aged over 40 years with end-stage kidney disease met the definition of frailty, which negatively affects their prognosis [44]. Ascorbate is considered an anti-aging factor owing to its antioxidant properties [3]. Therefore, ascorbate may be causally associated with life prognosis and aging in hemodialysis patients.

This study has several major limitations. First, the sample size was small. Second, there were no data concerning the oxidative stress levels in vivo. The third limitation was the lack of dietary survey. In future studies, we plan to improve these limitations.

## 5. Conclusions

Hemodialysis patients showed low plasma total ascorbate levels, including those of DHA; these levels were reduced by approximately 40% by a single hemodialysis. We found that hemodialysis notably increased the proportion of DHA in hemodialysis patients. The low plasma total ascorbate levels in these patients may be due to the decreased intake of ascorbate from fresh fruits and vegetables due to the strict restriction of potassium intake. To prevent ascorbate deficiency and the progression of aging by hemodialysis, hemodialysis patients should consume sufficient ascorbate from supplements or medicine because of the inability of the body to synthesize ascorbate.

## Figures and Tables

**Table 1:** Clinical characteristics of patients.

Characteristic	CKD Stage G3-G5 (*N = 27*)	Hemodialysis (*N = 19*)
Age (years)	83.9 ± 6.8	78.9 ± 10.9
Sex (male/female)	10/17	9/10
White blood cell (×10³/μL)	M: 6.7 ± 1.8	M: 6.5 ± 1.3
	F: 7.0 ± 1.7	F: 7.1 ± 3.5
Hemoglobin (g/dL)	M: 12.1 ± 1.2	M: 11.3 ± 0.6
	F: 11.8 ± 1.2	F: 11.2 ± 0.8
Hematocrit (%)	M: 36.5 ± 3.9	M: 34.3 ± 2.3
	F: 36.2 ± 3.0	F: 35.1 ± 2.5
Platelet (×10⁴/μL)	M: 20.5 ± 6.3	M: 23.8 ± 6.8
	F: 22.1 ± 5.8 *	F: 16.0 ± 6.0
Total protein (g/dL)	6.9 ± 0.5 *	6.3 ± 0.5
Albumin (g/dL)	3.6 ± 0.3 *	3.1 ± 0.5
C-reactive protein (mg/dL)	0.4 ± 0.7	0.8 ± 2.4
AST (IU/L)	21.2 ± 5.1 *	24.7 ± 36.4
**Blood urea nitrogen (mg/dL)**	32.9 ± 14.6 *	**62.7 ± 13.3**
**Creatinine (mg/dL)**	M: 2.0 ± 0.9 *	**M: 10.4 ± 1.8**
	F: 2.1 ± 1.0 *	**F: 7.4 ± 1.3**
Uric acid (mg/dL)	M: 6.0 ± 1.1	M: 5.9 ± 1.5
	F: 6.3 ± 1.7	F: 5.9 ± 1.6
Sodium (mEq/L)	140.4 ± 3.5 *	137.7 ± 3.6
Potassium (mEq/L)	4.4 ± 0.6	4.9 ± 0.8
Calcium (mg/dL)	9.1 ± 0.6 *	8.5 ± 0.5
Phosphorus (mg/dL)	3.8 ± 0.7 *	5.1 ± 1.3
Triglyceride (mg/dL)	150.9 ± 71.8 *	108.7 ± 32.8
Total cholesterol (mg/dL)	200.9 ± 47.8 *	152.8 ± 38.0
LDL cholesterol (mg/dL)	109.5 ± 34.7 *	81.2 ± 26.7
Iron (μg/dL)	M: 66.2 ± 13.8	M: 62.8 ± 44.7
	F: 76.6 ± 28.1 *	F: 47.5 ± 16.8
TIBC (μg/dL)	M: 234.6 ± 16.3	M: 251.7 ± 38.4
	F: 253.6 ± 35.5	F: 230.9 ± 48.9
Ferritin (ng/dL)	M: 159.6 ± 86.2	M:105.9 ± 83.5
	F: 177.2 ± 250.1	F: 117.3 ± 79.4
**β2-microglobulin (mg/L)**	5.1 ± 2.1 *	**27.0 ± 7.4**
Prealbumin (mg/dL)	23.2 ± 5.6	24.1 ± 5.2
HbA1c (%)	6.1 ± 0.7	5.8 ± 0.7

Values are presented as the mean ± SD. * Significant difference at *p* < 0.05. M—male; F—female; CKD—chronic kidney disease; AST—aspartate aminotransferase; LDL—low-density lipoprotein; TIBC—total iron-binding capacity; HbA1—hemoglobin A1c.

**Table 2:** Kendall’s rank correlation coefficient (τ) between clinical characteristics and total ascorbate, ascorbate, and dehydroascorbic acid (DHA) levels.

	Total Ascorbate			Ascorbate			DHA		
Characteristic	τ	*p*-Value	*q*-Value	τ	*p*-Value	*q*-Value	τ	*p*-Value	*q*-Value
Age	0.31	0.063	0.588	0.33	0.049 *	0.457	0.14	0.419	0.692
Dry weight	0.05	0.783	0.914	0.01	0.944	0.944	−0.02	0.916	0.916
White blood cell	−0.05	0.783	0.914	−0.05	0.779	0.922	−0.14	0.420	0.692
Hemoglobin	0.07	0.699	0.914	0.11	0.527	0.918	−0.22	0.193	0.600
Hematocrit	0.09	0.599	0.914	0.17	0.326	0.803	−0.21	0.220	0.611
Platelet	−0.20	0.238	0.680	−0.23	0.161	0.751	−0.28	0.100	0.400
Total Protein	0.08	0.647	0.914	0.02	0.888	0.922	0.10	0.548	0.797
**Albumin**	0.16	0.350	0.817	0.12	0.495	0.918	0.36	0.044 *	0.302
C-reactive protein	−0.19	0.261	0.680	−0.17	0.308	0.803	−0.29	0.092	0.400
AST	−0.19	0.260	0.680	−0.25	0.149	0.751	−0.06	0.725	0.872
Blood urea nitrogen	0.01	0.944	0.945	0.04	0.806	0.922	0.05	0.779	0.872
Creatinine	−0.09	0.629	0.914	−0.09	0.575	0.918	−0.10	0.552	0.797
Uric acid	−0.10	0.551	0.914	−0.12	0.483	0.918	−0.03	0.861	0.916
Sodium	0.13	0.458	0.914	0.12	0.480	0.918	0.20	0.243	0.611
**Potassium**	0.41	0.015 *	0.420	0.39	0.021 *	0.420	0.34	0.045 *	0.302
Calcium	−0.01	0.944	0.945	−0.04	0.805	0.922	−0.02	0.888	0.916
Phosphorus	-0.05	0.752	0.914	−0.08	0.623	0.918	−0.08	0.648	0.825
Triglyceride	0.19	0.263	0.680	0.16	0.344	0.803	0.19	0.262	0.611
Total cholesterol	0.19	0.248	0.680	0.18	0.293	0.803	0.33	0.054	0.302
LDL cholesterol	0.08	0.624	0.914	0.09	0.599	0.918	0.15	0.362	0.676
**Iron**	0.23	0.172	0.680	0.20	0.233	0.803	0.34	0.046 *	0.302
TIBC	−0.19	0.267	0.680	−0.19	0.263	0.803	−0.17	0.310	0.620
Ferritin	0.27	0.107	0.680	0.26	0.115	0.751	0.26	0.123	0.431
β2-microglobulin	−0.02	0.889	0.945	−0.03	0.861	0.922	−0.18	0.293	0.620
Prealbumin	−0.06	0.776	0.914	−0.07	0.711	0.922	0.10	0.592	0.797
HbA1c	−0.07	0.699	0.914	−0.07	0.673	0.922	−0.09	0.598	0.797
Parathyroid hormone	0.02	0.945	0.945	0.02	0.889	0.922	−0.05	0.753	0.872

* *p*-value < 0.05. AST—aspartate aminotransferase; LDL—low-density lipoprotein; TIBC—total iron-binding capacity; HbA1—hemoglobin A1c.

## Data Availability

Data are included in the text; raw data are available from the corresponding author.

## Appendix A

**Table A1:** Plasma total ascorbate, ascorbate, and dehydroascorbic acid (DHA) levels of the patients.

CKD stage G3–G5 (N = 27)		Total ascorbate	27.1 ± 13.9
		Ascorbate	18.0 ± 12.8
		DHA	9.1 ± 13.6
Hemodialysis (N = 19)	Pre-dialysis	Total ascorbate	12.0 ± 6.2
		Ascorbate	7.5 ± 4.8
		DHA	4.5 ± 1.8
	Post-dialysis	Total ascorbate	7.2 ± 3.9
		Ascorbate	3.2 ± 2.8
		DHA	4.0 ± 1.5

Values are presented as the mean (μM) ± SD.
